# Malignant priapism as the presenting manifestation of penile metastasis following radical cystoprostatectomy for bladder urothelial carcinoma: a case report

**DOI:** 10.1093/jscr/rjag631

**Published:** 2026-07-24

**Authors:** Reza Kaffash Nayeri, Farshad Banouei

**Affiliations:** Uro – Oncology, Department of Urology, Hasheminejad Kidney Center, School of Medicine, Iran University of Medical Sciences, Jeyhoon Street, Malek Street, Tehran 1348959111, Iran; Uro – Oncology Fellowship, Department of Urology, Hasheminejad Kidney Center, School of Medicine, Iran University of Medical Sciences, Jeyhoon Street, Malek Street, Tehran 1348959111, Iran

**Keywords:** priapism, penile metastasis, transitional cell carcinoma, bladder cancer, penectomy, ^18^F-FDG PET/CT

## Abstract

Penile metastasis is a rare but grave complication of systemic malignancies, occasionally manifesting as malignant priapism due to neoplastic infiltration of the corpora cavernosa. We report a 59-year-old male who presented with painful, refractory penile erection 3 years after radical cystoprostatectomy for bladder urothelial carcinoma (pT3bN0M0). Pelvic magnetic resonance imaging revealed a heterogeneous infiltrative mass involving the proximal corpora cavernosa bilaterally. ^18^F-fluorodeoxyglucose positron emission tomography/computed tomography confirmed multiple metabolically active penile lesions without distant metastasis. Core needle biopsy established the diagnosis of metastatic transitional cell carcinoma. Total penectomy was performed, and adjuvant chemotherapy was recommended. This case highlights the importance of clinical vigilance in urological malignancy survivors.

## Introduction

Priapism is defined as a prolonged penile erection persisting beyond four hours unrelated to sexual stimulation. While most cases are ischemic in etiology, malignant priapism—caused by neoplastic infiltration of the corpora cavernosa—is a rare and underrecognized variant [1].

Penile metastasis is an uncommon phenomenon despite the organ’s rich vascularity, with bladder cancer being the most frequently implicated primary malignancy, followed by prostate, colorectal, and renal carcinomas [2, 3]. Priapism occurs in ~20%–40% of penile metastasis cases due to venous occlusion from direct tumor infiltration [4].

Transitional cell carcinoma (TCC) of the bladder, particularly muscle-invasive disease, carries significant risk of recurrence following radical cystoprostatectomy, and may rarely recur at atypical sites, including the Fewer than 500 cases of penile metastasis have been reported worldwide, and those presenting as malignant priapism from bladder TCC are exceedingly rare. We present such a case to raise clinical awareness and highlight the diagnostic approach in this challenging entity [5].

## Case presentation

A 59-year-old male with a history of bladder TCC (pT3bN0M0) who had undergone radical cystoprostatectomy with ileal conduit urinary diversion three years prior was referred to the emergency department with a one-day history of progressively worsening penile pain and swelling. The erection had developed gradually without any preceding sexual stimulation, intracavernosal injection, or use of phosphodiesterase-5 inhibitors. Of particular relevance, the patient had noticed a small, asymptomatic nodule at the base of the penis ~6 months prior to this presentation, which he had not brought to medical attention. He denied fever, hematuria, dysuria, or other systemic symptoms. His past medical history included a 10-pack-year smoking history, and he was not on any regular medications.

On physical examination, a sustained, painful penile erection was evident. The entire penile shaft was swollen and erythematous, and the glans appeared dusky with restricted compressibility of the corpora cavernosa. There was no inguinal lymphadenopathy and no palpable abdominal or pelvic masses. Initial laboratory investigations, including complete blood count, coagulation profile, renal and hepatic function tests, and urinalysis, were within normal limits. Cavernosal blood gas analysis was not obtained; however, the clinical presentation—a rigid, painful erection with no response to initial conservative measures—was consistent with low-flow (ischemic) priapism. Intracavernosal injection of phenylephrine and corporeal aspiration were attempted without any detumescence, raising immediate suspicion for a malignant etiology.

Given the patient’s oncological background and failure of standard priapism management, urgent pelvic magnetic resonance imaging (MRI) was performed. This demonstrated a heterogeneous, infiltrative mass at the penile base with ill-defined margins, bilateral involvement of the proximal corpora cavernosa, and associated soft tissue edema, without bony involvement (Fig. 1A). To assess metabolic activity and systemic extent, ^18^F-fluorodeoxyglucose positron emission tomography/computed tomography (^18^F-FDG PET/CT) was subsequently obtained. This revealed multiple intensely FDG-avid lesions involving the penile base and shaft, strongly consistent with metastatic infiltration, with no hypermetabolic foci at other distant sites (Fig. 1B).

**Figure 1:**
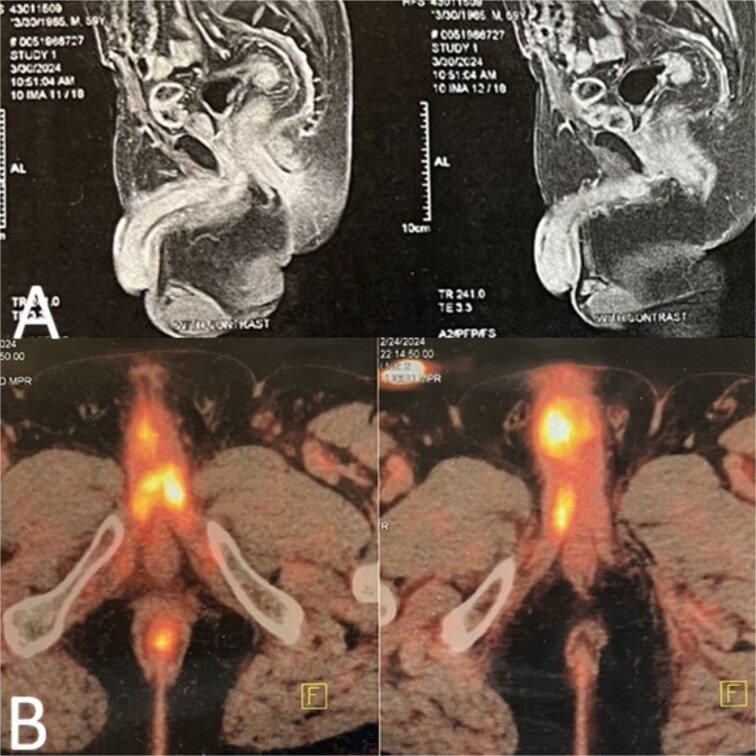
Imaging findings of metastatic penile lesion. (A) Pelvic MRI demonstrated a heterogeneously enhancing mass in the corpora cavernosa. (B) FDG avid lesion in the penile root and penile shaft consistent with metastasis.

Ultrasound-guided core needle biopsy of the penile mass was performed. Histopathological examination showed nests and sheets of atypical urothelial cells with high nuclear-to-cytoplasmic ratios and prominent nucleoli within a desmoplastic stroma. Immunohistochemical staining was positive for CK7, CK20, GATA3, and p63, confirming metastatic urothelial carcinoma consistent with the patient’s known bladder primary.

Following multidisciplinary team discussion, and given the refractory priapism, histologically confirmed metastatic penile involvement, and absence of distant disease on staging imaging, total penectomy with perineal urethrostomy was performed. The surgical specimen demonstrated extensive bilateral replacement of the corpora cavernosa by metastatic urothelial carcinoma with tumor-free surgical margins (Fig. 2). The postoperative course was uneventful. Adjuvant platinum-based chemotherapy was recommended following adequate recovery, and the patient was enrolled in a close oncological surveillance program.

**Figure 2:**
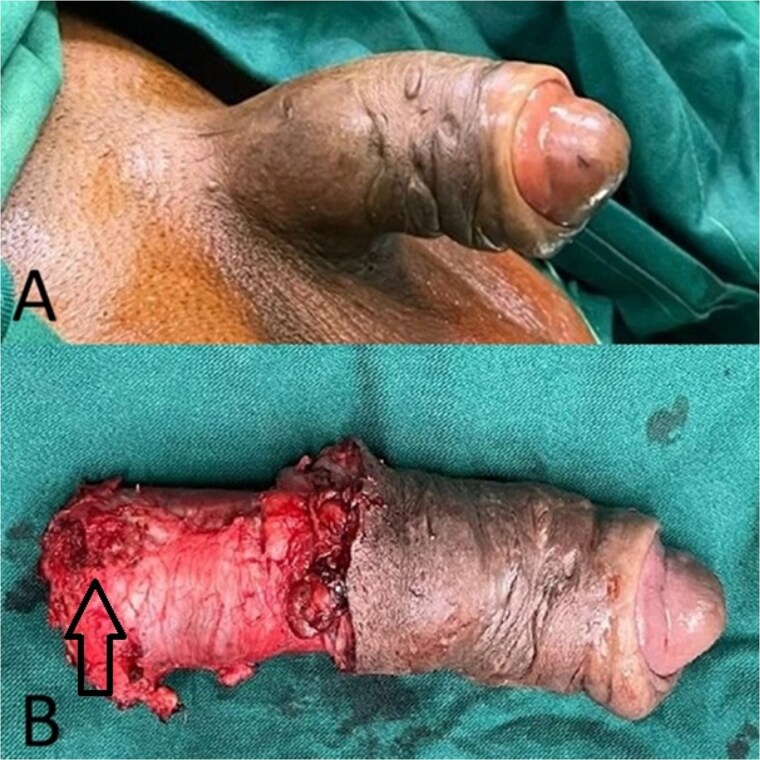
(A) Penile erection before surgery. (B) Metastasis in the proximal part of penis.

## Discussion

Penile metastasis is a rare complication of systemic malignancies, with bladder cancer accounting for ~29%–35% of documented cases [2, 3]. Metastatic routes include retrograde venous embolization via the prostatic venous plexus, direct extension, retrograde lymphatic spread, and arterial dissemination. In post-cystoprostatectomy patients, removal of the prostate may paradoxically facilitate retrograde venous flow toward the penile venous system, potentially contributing to metastatic seeding.

The six-month history of an unexamined penile nodule in our patient illustrates a critical diagnostic pitfall that underscores the necessity for improved patient education and surveillance strategies. Any new penile mass in a patient with a history of genitourinary malignancy warrants urgent evaluation. Healthcare providers—both urologists and primary care physicians—have a fundamental responsibility to counsel patients with a history of bladder, prostate, or other genitourinary malignancies about the potential for metastatic disease to present at unusual sites, including the penis. Patients should be educated to seek immediate medical attention at a clinic or emergency department if they notice any new penile lesions, nodules, or changes in penile appearance, persistent penile pain, or unexplained penile erection. This heightened awareness and proactive counseling could facilitate earlier diagnosis and potentially improve outcomes in this rare but devastating complication [6].

Overall prognosis remains poor. A comprehensive review by Chaux *et al.* documented median survival of 6–12 months in most series of penile metastasis; however, critical prognostic distinctions exist based on metastatic extent. Specifically, patients with isolated penile metastasis at diagnosis have demonstrated superior outcomes compared to those with concurrent distant metastases. In a subset analysis, cases confined to the penis without evidence of systemic dissemination at the time of diagnosis showed median survival extending to 12–18 months or beyond with combined multimodal therapy (penectomy with adjuvant chemotherapy), compared to 6 months or less in patients with synchronous distant disease. These favorable outcomes with isolated penile metastasis underscore the importance of aggressive locoregional treatment in appropriately selected patients [7].

## Data Availability

Data are available from the corresponding author on a reasonable request.
